# Expression of concern: Bone marrow mesenchymal stem cells inhibited bleomycin-induced lung fibrosis

**DOI:** 10.1039/d2ra90008d

**Published:** 2022-02-09

**Authors:** Laura Fisher

**Affiliations:** Royal Society of Chemistry Thomas Graham House, Science Park, Milton Road Cambridge CB4 0WF UK advances-rsc@rsc.org

## Abstract

Expression of concern for ‘Bone marrow mesenchymal stem cells inhibited bleomycin-induced lung fibrosis’ by Jiang Nan *et al.*, *RSC Adv.*, 2017, **7**, 49498–49504. DOI: 10.1039/C7RA03971A.

*RSC Advances* is publishing this expression of concern in order to alert readers that concerns have been raised over the integrity of the data published in this article. The authors have been contacted but have not responded to requests to provide raw data. An expression of concern will continue to be associated with the article until a conclusive outcome is reached.

Laura Fisher

31st January 2022

Executive Editor, *RSC Advances*

## Supplementary Material

